# Association between colorectal cancer and *Fusobacterium nucleatum* and *Bacteroides fragilis* bacteria in Iranian patients: a preliminary study

**DOI:** 10.1186/s13027-021-00381-4

**Published:** 2021-06-09

**Authors:** Aref Shariati, Shabnam Razavi, Ehsanollah Ghaznavi-Rad, Behnaz Jahanbin, Abolfazl Akbari, Samira Norzaee, Davood Darban-Sarokhalil

**Affiliations:** 1 Department of Microbiology, School of Medicine, Iran University of Medical Sciences, Tehran, Iran; 2 Microbial Biotechnology Research Center, Iran University of Medical Sciences, Tehran, Iran; 3 Department of Medical Laboratory Sciences, School of Allied Medical Sciences, Arak University of Medical Sciences, Arak, Iran; 4 Department of Pathology, Cancer Research Institute, Imam Khomeini Hospital Complex, Tehran University of Medical Science, Tehran, Iran; 5 Colorectal Research Center, Iran University of Medical Sciences, Tehran, Iran; 6 Research Center for Environmental Health Technology, Iran University of Medical Sciences, Tehran, Iran

**Keywords:** *Fusobacterium nucleatum*, *Bacteroides fragilis*, *Streptococcus bovis*/*gallolyticus*, Enteropathogenic *Escherichia coli*, Colorectal cancers, qPCR

## Abstract

**Background and aim:**

Recent studies have proposed that commensal bacteria might be involved in the development and progression of gastrointestinal disorders such as colorectal cancer (CRC). Therefore, in this study, the relative abundance of *Fusobacterium nucleatum*, *Bacteroides fragilis*, *Streptococcus bovis*/*gallolyticus*, and Enteropathogenic *Escherichia coli* (EPEC) in CRC tissues, and their association with clinicopathologic characteristics of CRC was investigated in Iranian patients. Moreover, the role of these bacteria in the CRC-associated mutations including *PIK3CA*, *KRAS*, and *BRAF* was studied.

**Method:**

To these ends, the noted bacteria were quantified in paired tumors and normal tissue specimens of 30 CRC patients, by TaqMan quantitative Real-Time Polymerase Chain Reaction (qPCR). Next, possible correlations between clinicopathologic factors and mutations in *PIK3CA*, *KRAS*, and *BRAF* genes were analyzed.

**Results:**

In studied samples, *B. fragilis* was the most abundant bacteria that was detected in 66 and 60% of paired tumor and normal samples, respectively. Furthermore, 15% of the *B. fragilis*-positive patients were infected with Enterotoxigenic *B. fragilis* (ETBF) in both adenocarcinoma and matched adjacent normal samples. *F. nucleatum* was also identified in 23% of tumors and 13% of adjacent normal tissue samples. Moreover, the relative abundance of these bacteria determined by 2^-ΔCT^ was significantly higher in CRC samples than in adjacent normal mucosa (*p* < 0.05). On the other hand, our findings indicated that *S. gallolyticus* and EPEC, compared to adjacent normal mucosa, were not prevalent in CRC tissues. Finally, our results revealed a correlation between *F. nucleatum*-positive patients and the *KRAS* mutation (*p* = 0.02), while analyses did not show any association between bacteria and mutation in *PIK3CA* and *BRAF* genes.

**Conclusion:**

The present study is the first report on the analysis of different bacteria in CRC tissue samples of Iranian patients. Our findings revealed that *F. nucleatum* and *B. fragilis* might be linked to CRC. However, any link between gut microbiome dysbiosis and CRC remains unknown.

## Introduction

Colorectal cancer (CRC) is identified as one of the leading causes of morbidity and mortality around the world. It is known as the third most prevalent cancer in Iran and its mortality rate has been growing in recent years [1–3]. In the 1996–2000 period, the incidence rate of CRC was 7–8 per 100,000 for both males and females in Iran [4], while this rate increased to 11.8 and 16.5 (per 100,000) for females and males, respectively, in 2014 [5]. The World Health Organization (WHO) reported in 2014 that the CRC incidence rate, particularly among men, had been rapidly increased in the past decades in Eastern Europe and Asia, including Iran [6].

Hereditary forms, such as familial adenomatous polyposis and hereditary non-polyposis colon cancers, constitute less than 5% of CRCs, while the majority are sporadic cases caused by factors such as environmental exposures and lifestyle. CRC is a multistep carcinogenic disease and recent studies have reported that the gut microbiome may have a key role in colorectal tumorigenesis [2, 7]. Furthermore, CRC is related to oxidative processes and chronic inflammation which may induce malignant cell transformation and trigger carcinogenic processes like angiogenesis and proliferation [8].

Besides, a high prevalence of infectious agents such as *Fusobacterium nucleatum*, *Bacteroides fragilis*, *Streptococcus gallolyticus,* and Enteropathogenic *Escherichia coli* (EPEC), in tissue or fecal samples of CRC patients has been reported [9–12]. Based on different observations, bacterial-driven oncogenic mechanisms such as B catenin/Wnt signaling in *F. nucleatum*, EPEC, and *B. fragilis*, pro-inflammatory signaling in *S. gallolyticus*, and genotoxicity in EPEC may be involved in the development of CRC [9]. Also, specific gut bacteria might be related to a specific molecular carcinogenesis pathway in the colorectal. In this respect, the association between the high concentration of *F. nucleatum* and mutation in *BRAF* and *KRAS* genes has been reported [13, 14]. Recent studies have examined the association of different bacteria with different cancers such as CRC; however, the reports are different [15]. The association of bacteria with CRC has been widely investigated in Europe and North America where the genetic and ethnic traits of patients differ from those in Asia, and this could influence the gut microbial composition [16].

The association between multiple bacteria and CRC has not been investigated in Iran. Hence, the current research employs TaqMan quantitative Real-Time Polymerase Chain Reaction (qPCR) to detect the presence and abundance of four highly CRC-associated bacteria (*F. nucleatum*, *B. fragilis*, EPEC, and *S. gallolyticus*) in CRC and adjacent nontumor tissues. It also investigates the association between these infectious agents and the molecular and clinicopathologic features of CRC.

## Materials and methods

### Sample preparation

**Fig. 1:**
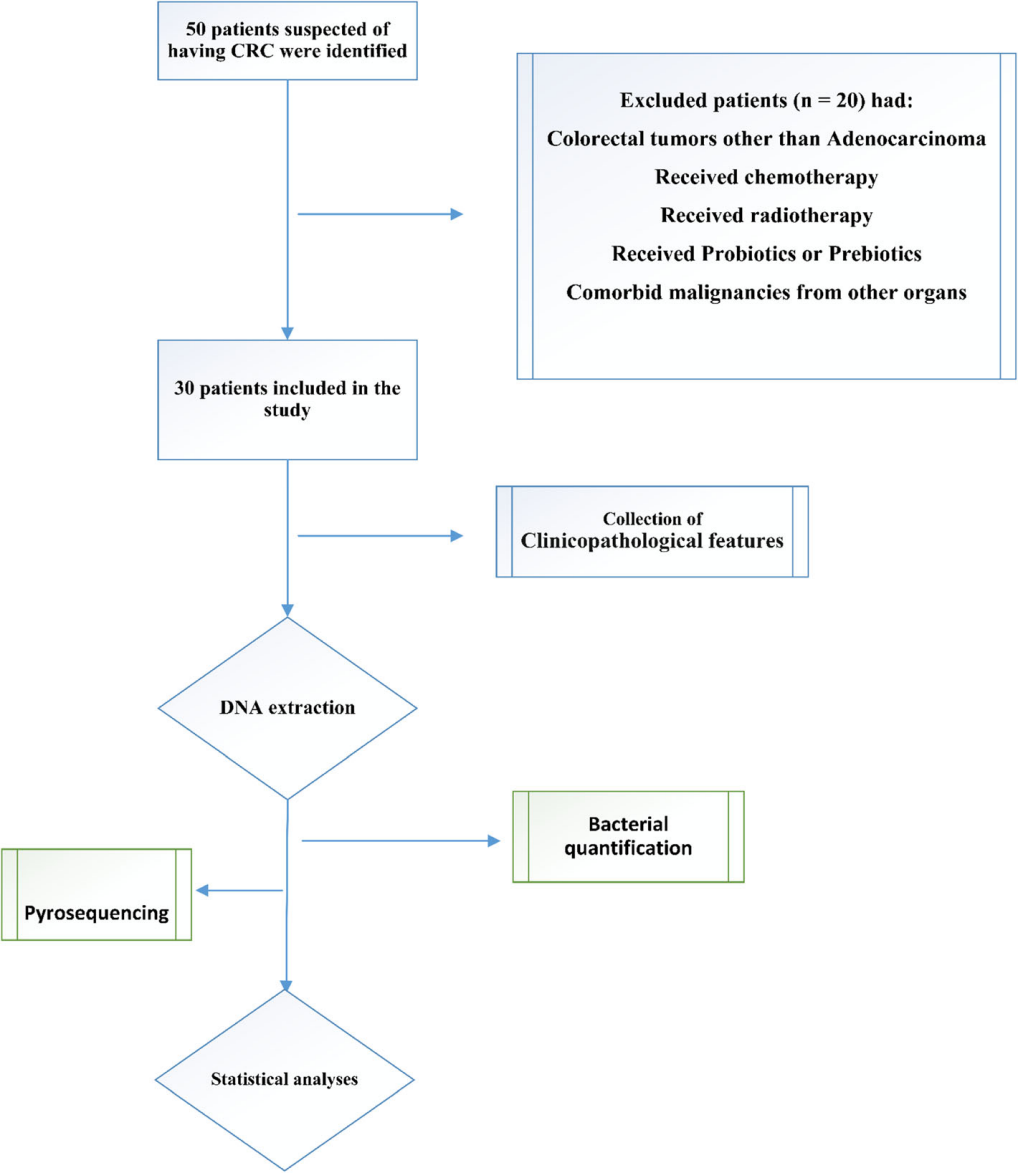
A schematic diagram of the experimental design from the biopsy collection to statistical analyses

In the present cross-sectional study, colorectal adenocarcinoma and adjacent normal tissues were randomly obtained from the patients who had visited the Hazrat-e Rasool General Hospital and Imam Khomeini hospital in Tehran between February 2019 and January 2021. The study protocols were approved by the Ethics Committee of the Iran University of Medical Sciences. All biopsies were collected following resection of the primary tumor or prior to any treatment regime. Participants were enrolled in the research before the surgery, and informed consent was obtained from all patients. After arterial ligation and surgical resection of the tissues, the samples were quickly transferred from the operating suite to the pathology unit where they were assessed by the pathologist who was blind to the clinical and molecular information. In addition, one part of the control mucosa samples and one of the tumorous tissues were chosen and fixed in RNAlater Reagent (QIAGEN, Hilden, Germany). They were then snap-frozen and stored at − 70 °C until DNA extraction. All clinical data and required information such as gender, age, and histopathological parameters were captured from patients’ records and case report forms. Of note, the exclusion criteria included patients who had (a) colorectal tumors other than adenocarcinoma, (b) antibiotics, probiotics, radiotherapy, or chemotherapy before surgery, and (c) comorbid malignancies from other organs (Fig. 1) [17].

### DNA extraction

Genomic DNA was extracted directly from the CRC and adjacent normal mucosal tissues (25 mg of each tissue) using QIAamp DNA Mini Kit (QIAGEN, Hilden, Germany). Upon extraction, DNA quantity and quality were analyzed at OD (260) and on an agarose gel. Then, verified DNA extracts were preserved at − 20 °C for subsequent analyses and qPCR.

### Real-time PCR

Custom TaqMan primer-probe sets were designed to detect the 16S rDNA gene sequence of *B. fragilis* and *F. nucleatum*. Furthermore, the primer-probe set for EPEC and *S. gallolyticus* was designed to target *eaeA* and *sodA* genes of these bacteria, respectively (Table 1). The reference gene, *SLCO2A1*, was used for normalization of the cycle threshold (CT) values of each bacterium, as a previous study [19]. Noteworthy, PCR assay for the detection of *B. fragilis* enterotoxin gene (*bft*) is carried out as explained by Kouhsari et al. [20]. In this regard, *B. fragilis* ATCC 43858 and ATCC 25285 strains were used as positive and negative controls, respectively.

**Table 1:** Utilized primers and TaqMan probes in the present research

Target bacteria	Primer/Probe	Oligonucleotide sequence (50e30)	Size (bp)	Product size (bp)	Ref
*Streptococcus gallolyticus subsp. gallolyticus*	Primer F Primer R Probe	CAGAAATTGGAGAGGATTTGGAAG CCGTTATTAATCACCGCTTGAC ATATCTGCTGGAATACTGTCAACATCTGCC	24 22 30	85	This study
EPEC	Primer F Primer R Probe	AGACTACAGGAAAATCGCTTGTTAGC CGTCCCTTTGATTCCAGTTCC TTCAACATTACCGTCATCAATAGCAAGTGG	26 21 30	140	This study
*Fusobacterium nucleatum*	Primer F Primer R Probe	AGCTACAAGAGAAGAAAATGAAAATGG CCAACTCCTACAAATCCAGTAACC TTACTTCATACCATACACGAGGATCTACTT	27 24 30	105	This study
*Bacteroides fragilis*	Primer F Primer R Probe	CGAGGGGCATCAGGAAGAA CGGAATCATTATGCTATCGGGTA CTTGCTTTCTTTGCTGGCGACCG	19 23 23	136	[18]
*SLCO2A1*	Primer F Primer R Probe	GAGAGATTTGAATGTTGGACAAAGC ACACTTCTGTGGTCACTCGTC TCCTACTGCCATCCTTCTACCTGCCA	25 21 26	89	This study

The specificities of the primers and probes were tested by Allele ID software (v.7.5) and the EMBL-EPI and NCBI BLAST databases. Note that in CRC cases with detectable bacteria, the CT values in the qPCR for each bacterium and *SLCO2A1* decreased linearly in proportion to the amount of input DNA (in a log scale) of the same specimen (r^2^ > 0.99). Besides, all assays were carried out in duplicate in a single patch and the results were averaged, and thus the reported data in this article are the mean values of duplicate qPCR analyses. Each reaction mixture contained 0.5 μM of each primer, 0.25 μM of the probe, 20 ng of extracted DNA, and 9 μl of Universal Probe Ex Taq PCR Master Mix (Ampliqon, Denmark), in a total volume of 20 μl. Furthermore, qPCR was conducted by Rotor-Gene 6000 real-time PCR cycler (Qiagen Corbett, Hilden, Germany) using the following thermocycling program: an initial holding at 95 °C for 15 min, followed by 40 cycles of denaturation at 95 °C for 15 s, and annealing/extension at 62 °C for 30 s. All the components of the reaction mixture without genomic DNA were used as a negative control in all analyses. The prevalence of four bacteria in every sample was assessed as a relative unitless value normalized to *SLCO2A1* using the 2^-ΔCT^ method (where ΔCT is the difference in the average CT value of each bacterium and the reference gene), as described previously [17, 19].

Finally, bacterial standard strains used in the present investigation were *F. nucleatum* ATCC 25586, *B. fragilis* ATCC 43858, *S. bovis* subsp. *gallolyticus* IBRC-M 10637, and *Escherichia coli* EPEC (M) O55: K 59 PTCC 1269.

### Pyrosequencing

Pyrosequencing of *BRAF* (codon 600), exon 3 of *KRAS* (codons 12 and 13), and *PIK3CA* (exon 9) was performed using the PyroMark Q96 ID QIAGEN software 2.5 system according to the manufacturer’s manual. For pyrosequencing, we used specific primers one of which was biotinylated to immobilize with streptavidin beads (GE healthcare) and to amplify each target region. The pyrosequencing reaction applied to Roche PCR 480 contained 20 ng of genomic DNA and results were analyzed by PyroMark Q24 Application Software 2.0 (v.2.0.6, Qiagen, Hilden, Germany). Finally, the results were double-checked by a High-Resolution Melting (HRM) analysis [21].

### Statistical analyses

To test the relative quantities of each bacterium between paired tumor and adjacent normal mucosa, we performed a Wilcoxon signed-rank test and applied it to a subset of specimens. Also, to evaluate the relationship between the ordinal (positive or negative) categories of the number of bacteria and categorical data, a Fisher exact test was employed. Statistical analyses were performed using GraphPad Prism v.8.3.0 and SPSS v.20.0 software (SPSS Inc. Chicago, IL, USA). A two-tailed *p*-value below 0.05 was considered statistically significant.

## Results

### Clinicopathological features of CRC patients in Iran

The histopathologic and demographic properties of patients are shown in Table 2. Briefly, a total of 20 men and 10 women with an average age of 57 (SD ± 11.04, range 26–76) were included in the present study. 73.3% of patients were struggling with grade-II (moderately differentiated) cancer, and 13.3, 6.3, and 3.3% of them respectively with grade I (well-differentiated), grade III (poorly differentiated), and grade IV (undifferentiated). Noteworthy, there was only one patient (3.3%) with Grade-X cancer (Table 2). The cohort consisted of 60% colon and 40% rectal cancers, with proximal cancers accounting for 30% of the colon cancers. Finally, only 3% of the patients were alcohol drinkers, while 23.3% of them were smokers.

**Table 2:** Clinicopathological features of the patients in the present study (2019–2021, Number = 30)

**Clinicopathological Characteristics of CRC Patients**	
Male/Female (n (%))	20 (66.7%)/10 (33.3%)
Age: Mean ± SE	57 (57 ± 11.04)
**Site of primary**	
Cecum	10%
Ascending colon	6.7%
Hepatic flexure	3.3%
Transverse Colon	6.7%
Splenic Flexure	3.3%
Descending Colon	3.3%
Sigmoid Colon	6.7%
Rectosigmoid	6.7%
Rectum	40%
Colon, NOS	13.3%
**Tumor size**	
Size ≤5	53.3%
Size > 5	46.7%
**Grade**	
I: (Well Differentiated)	13.3%
II: Moderately Differentiated	73.3%
III: Poorly Differentiated	6.7%
IV: Undifferentiated	3.3%
X: Unknown	3.3%
**Invasion, Nodal status, and Tumor deposit**	
Lymphatic	90%
Perineural	60%
Perineal	6.7%
Extramural Blood Vessel	20%
Extra-Nodal Extension	6.7%
Perforation	3.3%
Peritoneal Seeding	3.3%
**TNM staging**	
Stage I	6.7%
Stage IIA	50%
Stage IIIB	16.7%
Stage IIIC	13.3%
Stage IV	13.3%

### Bacterial quantification

**Fig. 2:**
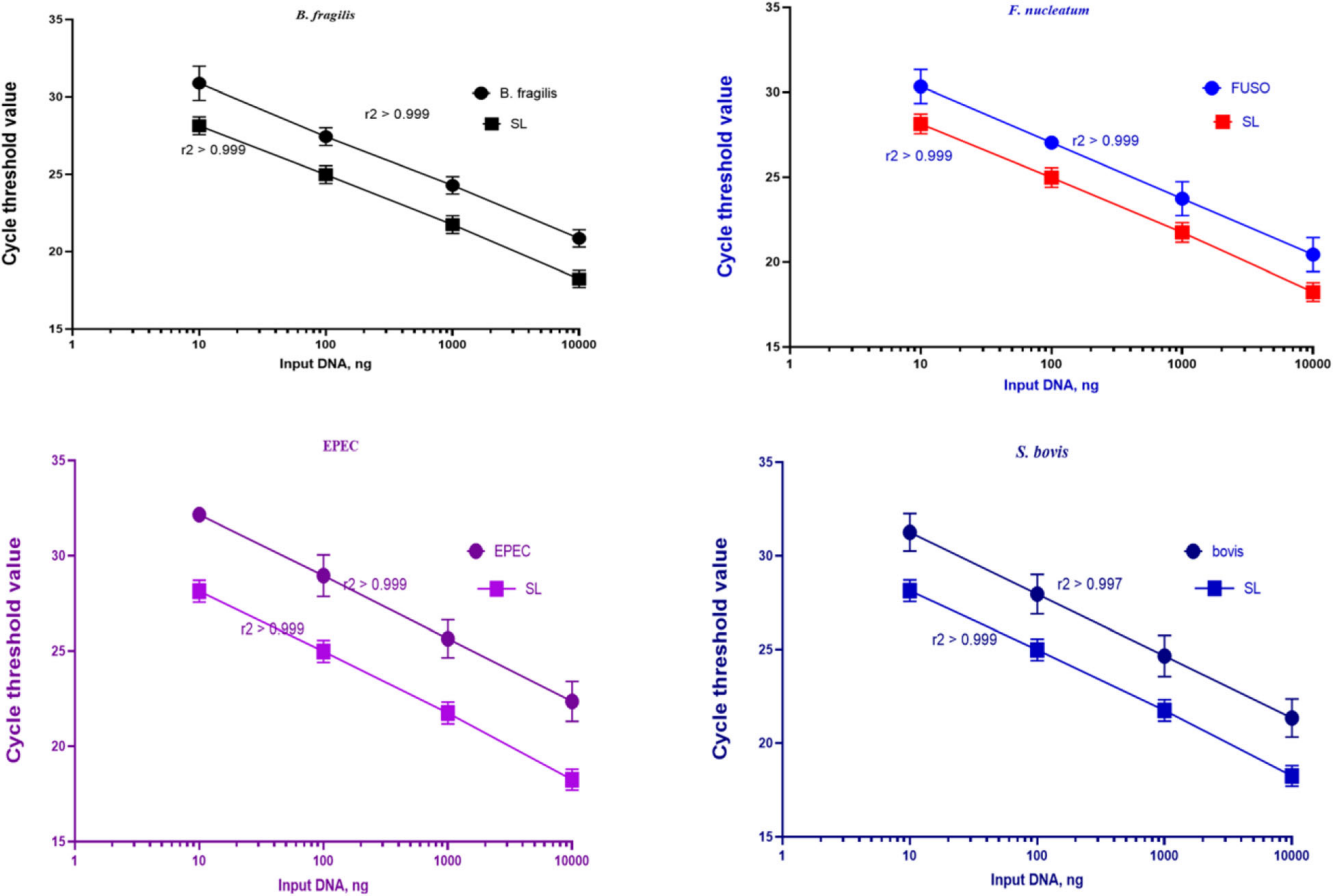
Quantitative Real-time Polymerase Chain Reaction (qPCR) assays for bacteria and the human reference gene (SLCO2A1) were carried out using 10-fold dilution series from the same DNA samples. Findings were conducted with triplicate runs in three separate examinations, and symbols showed mean, error bars, and standard deviation of cycle threshold values of triplicate runs. The coefficient of determination (r^2^) in the assays for each bacterium and SLCO2A1 is shown

**Fig. 3:**
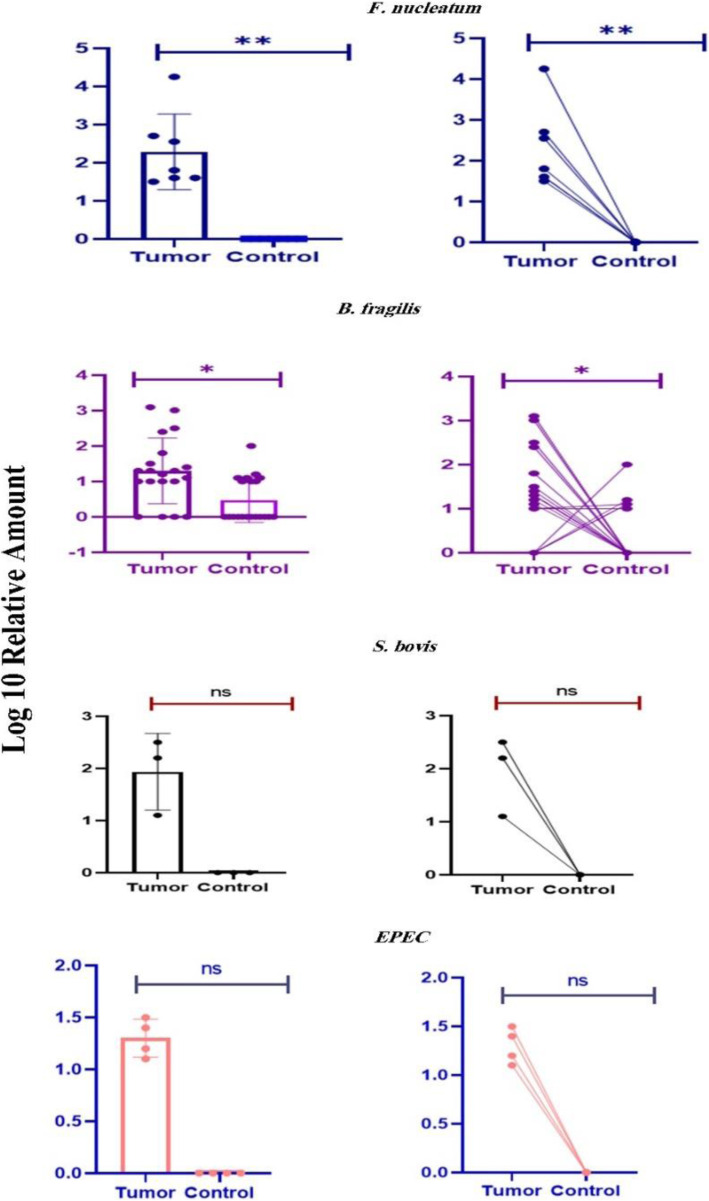
Relative quantification of the bacteria in CRC and adjacent normal mucous tissues. Scatter plots with bar (left) and relative amount of each bacterium in tumor and matched adjacent normal samples (right) are illustrated. The relative quantity of *F. nucleatum* (*n* = 30, *p* < 0.01**) and *B. fragilis* (*n* = 30, *p* < 0.05*) was significantly higher in CRC tissues than the adjacent normal mucosa that was collected 10–15 cm beyond cancer margins. On the other hand, there was no significant difference in the relative quantification of EPEC and *S. gallolyticus* (*n* = 30, *p* > 0.05) between CRC and non-CRC tissues

We considered high linearity (r^2^ > 0.99) and reproducibility (interassay coefficient of variation ≤1%) for validation of the qPCR assessment for each bacterium in CRC tissue specimens (Fig. 2). 30 CRC tissues and their adjacent normal mucosa were assessed for *F. nucleatum*, and seven CRC (23%) and four non-CRC (13%) tissues were found positive for bacterial DNA. In other words, in four samples, *F. nucleatum* was identified in both the lesion and matched normal mucosa. Moreover, the analysis of the relative abundance of *F. nucleatum* in CRC tissues was conducted in a paired way by the adjacent normal mucosa of each sample as its specific calibrator. Findings were consistent with the unpaired analysis results, which showed a larger quantity of *F. nucleatum* in CRC tissues (40–10^4^-fold, *p* < 0.01, Wilcoxon signed-rank test) (Fig. 3). None of these patients had a history of cancer in their family, and all were over 50 years old. Besides, one CRC sample had a higher concentration of bacteria compared to the matched normal mucosa (10^4^-fold). This sample belonged to a patient who had the largest lesion size (9 cm) among all studied patients. Furthermore, peritoneal seeding was observed only in this patient.

Among four bacteria, *B. fragilis* had the highest frequency and was detected in 20 (66%) CRC and 18 (60%) adjacent nontumor tissues. Noteworthy, unlike *F. nucleatum*, the median abundance of *B. fragilis* determined by 2^-ΔΔCT^ was significantly greater in five normal samples than in the adjacent tumor tissues (4–100-fold). Besides, in three samples, an equal quantity of *B. fragilis* was observed in both tumor and control samples. However, statistical analyses showed that the concentration of this bacterium was significantly higher in CRC tissues than in control samples (12–1024-fold, *p* < 0.05, Wilcoxon signed-rank test). In addition, stage-IV cancer and a tumor of 8 cm in size were recognized in one sample containing a high concentration of *B. fragilis* in CRC tissue, compared to the paired normal mucosa (1024-fold). Furthermore, out of the 20 tumor tissues and 18 adjacent normal samples that were positive for *B. fragilis*, respectively 5 (25%) and 3 (16%) turned out to harbor the *bft* gene (Enterotoxigenic *B. fragilis*, ETBF). In this manner, 15% of the *B. fragilis*-positive patients were infected with ETBF in both adenocarcinoma and matched adjacent normal samples, while in two patients ETBF was just detected in tumor tissue. DNA sequencing also confirmed these findings, and statistical analyses did not show significant differences for ETBF relative abundance between tumors and normal samples (*p* > 0.05).

Moreover, *S. gallolyticus* was detected in three (10%) and one (3%) paired tumor and normal samples, respectively. We identified EPEC via the Intimin gene (*eaeA*) in four (13%) colorectal adenocarcinomas and one (3%) corresponding normal mucus. Interestingly, no variation was detected for *S. gallolyticus* and EPEC between tumor tissues and normal mucosa samples of CRC patients (*p* > 0.05). Notably, our results did not show any significant clinical associations between *S. gallolyticus* and EPEC colonization. This is not surprising given the small number of *S. gallolyticus*- and EPEC-positive patients. Altogether, no bacteria were detected in 43.3% of the CRC samples, while more than one bacterial species was identified in 23.3% of the CRC biopsies.

### Clinicopathological and molecular characteristics of CRC patients and their bacterial status

In this study, no considerable correlation between the four surveyed bacteria and CRC clinicopathological variables such as tumor stage and location, pathological differentiation, and infiltration depth was observed (*p* > 0.05). Notably, the concentration of *F. nucleatum* was higher in the rectum than in the colon. Furthermore, pathologic data about all *B. fragilis*-positive patients revealed lymphovascular invasion; however, statistical analyses did not show any correlation (*p* > 0.05) between the bacterium and the mentioned markers.

We also studied CRC-related mutation. *KRAS* (codons 12 and 13) mutations were identified in 30% of cases, while 60 and 30% of these cases experienced mutations in codons 12 and 13, respectively. Besides, the most commonly detected mutation in codon 12 was Gly12Asp, followed by Gly12Val and Gly12Ser. Moreover, the *F. nucleatum*-positive CRC patients had *KRAS* mutations more frequently than *F. nucleatum*-negative CRC patients (*p-value* = 0.02) (Table 3). In contrast, we did not find any *PIK3A* mutation in the samples, and *BRAF* mutation was detected in only 2 (6.6%) specimens. Finally, there was no significant correlation between these mutations and bacterial colonization.

**Table 3:** Correlation between bacterial population, clinicopathological parameters, and molecular features

Spearman’s rho	***F. nucleatum***	***B. fragilis***	***S. gallolyticus***	EPEC
Age	0.18	0.21	0.31	0.33
Sex	0.09	0.12	0.25	0.54
Tumor location	0.06	0.25	0.48	0.51
Tumor size	0.07	0.13	0.25	0.31
Disease stage				
Tumor infiltration	0.19	0.25	0.30	0.52
Lymphatic metastasis	0.11	0.08	0.29	0.46
TNM staging	0.11	0.25	0.31	0.41
*KRAS* mutation	0.02*	0.19	0.54	0.25
*BRAF* mutation	0.10	0.30	0.91	0.86
*PIK3CA* mutation	0.12	0.48	0.88	0.91

(*P value < 0.05. *EPEC* Enteropathogenic *E. coli*)

## Discussion

In this study, the abundance of different bacteria was analyzed in fresh frozen biopsies of colorectal lesions of 30 CRC patients, and the quantities were compared to their adjacent non-CRC tissue. Furthermore, we studied the association between the bacteria and the corresponding clinicopathological and molecular characteristics. In previous research, healthy controls without colorectal neoplasia were employed as a control sample [22]; however, here, adjacent normal mucosal samples of CRC patients were used, as it is a common approach to attenuate the effect of genetic background [23]. Moreover, different pieces of research have used Formalin-Fixed Paraffin-Embedded (FFPE) tissue and fecal samples instead of fresh frozen biopsies to investigate the association between bacterial pathogens and CRC [19, 22]. Nevertheless, we analyzed fresh frozen tissues. FFPE, due to the formation of crosslinks, interferes with the analysis of biomolecules, especially nucleic acids [24]. In addition, stool specimens can only partially exhibit the mucosal bacterial composition in patients with CRC, and Gevers et al. reported that the microbiome diversity, such as *Fusobacterium* spp., could only be detected in the tissue specimens rather than in stool samples collected during diagnostic processes [2, 25]. Therefore, in the current research, the TaqMan qPCR assay was employed to measure the fold-change of bacteria in CRC tissue and adjacent normal mucosal samples.

According to our findings, *F. nucleatum* was highly abundant in 23% of CRC tissues compared to adjacent normal mucosa. *F. nucleatum* employs different virulence factors such as FadA and Fap2 to reinforce its binding to E-cadherin, and consequently to activate the B-catenin pathway and instigate an immune-mediated inflammation. Hence, this bacterium induces an inflammatory and oncogenic response that may lead to the initiation and progression of CRC [8, 26]. Consistently, various research studies have reported a positive correlation between *F. nucleatum* and this cancer. However, *F. nucleatum* abundance in patients with CRC varied between 13 and 87% in different countries [15, 19, 22, 27]. Given that the gut microbiome can differ from person to person, some factors such as weight, body mass index, diet, and geographical location may play an active role in bringing about this variation. Besides, using different detection techniques such as various real-time methods, pyrosequencing, Fluorescence In Situ Hybridization (FISH), and detection-specific antibodies, as well as application of diverse samples like FFPE, fresh frozen tissues, and stools can be effective in the inconsistency of reports [15, 23]. In Iran, investigations to determine the role of *F. nucleatum* in CRC are missing. Unlike our observations, Kashani et al. reported that 68% (24/35) and 24% (11/45) of Iranian CRC and non-CRC patients were colonized by *F. nucleatum*, respectively [28]. A higher degree of observed *F. nucleatum* colonization in CRC patients in this study compared to ours might be due to technical differences between simple PCR and qPCR. Another quantification report in Iran with SYBR qPCR assay and on stool samples reported a greater prevalence of *F. nucleatum* in the tubular adenoma and villous/tubulovillous polyp, than normal samples [29]. Therefore, as long as false positives or negatives may be reported in different research studies, it is strongly recommended that standard methods be used to detect the prevalence of *F. nucleatum*.

Here, we also detected *B. fragilis* in 66 and 60% of CRC and nontumor tissues, respectively, and statistical analyses showed a significantly higher prevalence rate of this bacterium in CRC tissues (*p* < 0.05). Furthermore, 15% of the *B. fragilis*-positive patients were infected with ETBF in both adenocarcinoma and matched adjacent normal samples. This finding is in agreement with other reports in Iran on mucosal biopsy samples, which reported *B. fragilis* in 63% (43/68) and 81% (42/52) of CRC patients and healthy controls, respectively [11]. Furthermore, the authors detected the *bft* gene in 47% of patients and 3.8% of the healthy control samples and showed that the level of *B. fragilis* and the difference between the positivity of the *bft* gene in CRC patients and healthy control was statistically significant. A recent investigation in Iran identified *B. fragilis* in 58.3 and 26.6% of the CRC patients and healthy volunteer stool samples. The *bft* gene was also detected in 31.6% of CRC cases, compared with only 8.3% in healthy controls [30]. Furthermore, Viljoen et al. detected ETBF in 26% (14/54) of colorectal adenocarcinomas and 28% (15/53) of adjacent normal mucosa samples in South Africa [9]. Another study also reported a higher number of ETBF in the stool of Iranian patients with tubular adenoma and villous/tubulovillous polyp in contrast to the normal controls [29]. It is assumed that enzymatically-active protein toxins such as BFT and *F. nucleatum* Adhesin A (FadA) can either directly induce host cell DNA damage or interfere with essential host cell signaling pathways involved in inflammation, cell proliferation, and apoptosis. In general, different bacteria with their protein virulence factors can affect host cell integrity, induce mutations and genome instability, and manipulate host cell signaling pathways. These effects can modulate cell proliferation, replication, and death, and coincidentally cause transformation and cellular malignancies. In this regard, evaluating the presence of bacterial toxins with oncogenic potential at the transcriptional or proteomic level will provide an additional layer of information to unravel complex host-pathogen interactions with relevance to CRC in the future [9, 31].

Collectively, the high frequency of *B. fragilis* in CRC and adjacent normal samples illustrates the fact that changes in bacterial composition might be linked to the transformation of colorectal mucosa from early adenomatous polyp stages to the latest CRC stages. By contrast, changes in the gut microbiota could be the consequence of CRC [16]. Nonetheless, these hypotheses require further research to verify the possible association between CRC and the high concentration of bacteria. As mentioned, ETBF is found in a higher amount in samples of CRC patients than of healthy individuals. On the other hand, based on our results and recent investigations, *B. fragilis* without toxin gene is also present in tumor tissues and the exact role of this bacterial species in the development and progression of CRS needs to be investigated.

We also indicated that *S. gallolyticus* and EPEC, compared to the adjacent normal mucosa, were not significantly prevalent in CRC tissues of Iranian patients. These results were in line with the research performed by Viljoen et al. who did not detect *S. gallolyticus* in any of the CRC or matched normal mucosa specimens [9]. They also reported a low number (6/54, 11%) of EPEC-positive patients in their cohort. In addition, in another research in Spain, only six out of 190 (3.2%) CRC patients were detected by qPCR to be positive for *S. gallolyticus* [32]. On the other hand, a recent investigation in Iran has detected this bacterium in 40% (9/22) and 5% (2/40) of stool cases in CRC patients and healthy controls. This study employed simple PCR and nonspecific primers as markers [10]. In another examination in China, no significant abundance of EPEC in CRC tissue and adjacent normal mucous biopsies was identified [16]. Therefore, inconsistent reports could originate from ethnic differences in susceptibility to bacterial colonization as well as employment of varied techniques.

Recent studies have reported that the interactions between genetic and epigenetic factors are involved in the tumorigenesis of the bacteria such as *F. nucleatum*. Accordingly, our findings revealed that there was a significant correlation between *F. nucleatum* and *KRAS* mutation. This result was consistent with a recent cohort study that reported a correlation between the abundance of this bacterium with *KRAS* mutation, in Japanese CRC patients [14]. Nevertheless, previous research did not find such a correlation [19, 27]. A relationship between the high concentration of *F. nucleatum* and *BRAF* V600E mutation was also reported, while such an association was not reported for *KRAS* [13]. This discrepancy could result from the different methods used for evaluating the number of bacteria and different cutoff values [14]. Furthermore, since we included only a small number of CRC patients with mutant genes (*n* = 11), more investigations would be required to analyze these correlations.

This study was subject to a number of limitations and constraints. First, the sample size used in this investigation was quite small. Second, due to the limited scope of sufficient data on the follow-up, we could not assess the bacteria for a longer period to relapse. In the end, we could not analyze the bacterial concentration/number in the normal colorectal tissue of healthy subjects because we did not use healthy volunteers for biopsy due to ethical issues.

## Conclusion

Our analyses showed a significantly higher abundance of *F. nucleatum* and *B. fragilis* bacteria in CRC samples compared to normal tissues; however, we could not detect such a relation for *S. gallolyticus* and EPEC. Due to diverse reports by different research groups, it is recommended that the role of each CRC-associated bacteria with CRC be further investigated in vivo and in vitro. In-depth research may facilitate organizing novel approaches to the diagnosis, prevention, and treatment of CRC.

## Data Availability

Data sharing not applicable to this article as no datasets were generated during the current study.
